# Effects of Patent Ductus Arteriosus on the Hemodynamics of Modified Blalock–Taussig Shunt Based on Patient-Specific Simulation

**DOI:** 10.3389/fphys.2021.707128

**Published:** 2021-08-31

**Authors:** Jiwen Xiong, Qi Sun, Yi Qian, Liwei Hu, Zhirong Tong, Jinfen Liu, Jinlong Liu

**Affiliations:** ^1^Department of Cardiothoracic Surgery, Shanghai Children's Medical Center, School of Medicine, Shanghai Jiao Tong University, Shanghai, China; ^2^Australian School of Advanced Medicine, Macquarie University, Sydney, NSW, Australia; ^3^Diagnostic Imaging Center, Shanghai Children's Medical Center, School of Medicine, Shanghai Jiao Tong University, Shanghai, China; ^4^Shanghai Engineering Research Center of Virtual Reality of Structural Heart Disease, Shanghai Children's Medical Center, School of Medicine, Shanghai Jiao Tong University, Shanghai, China; ^5^Institute of Pediatric Translational Medicine, Shanghai Children's Medical Center, School of Medicine, Shanghai Jiao Tong University, Shanghai, China

**Keywords:** modified Blalock-Taussig shunt, patent ductus arteriosus, computational fluid dynamics, hemodynamics, virtual surgery

## Abstract

The question of preserving the patent ductus arteriosus (PDA) during the modified Blalock–Taussig shunt (MBTS) procedure remains controversial. The goal of this study was to investigate the effects of the PDA on the flow features of the MBTS to help with preoperative surgery design and postoperative prediction. In this study, a patient with pulmonary atresia and PDA was included. A patient-specific three-dimensional model was reconstructed, and virtual surgeries of shunt insertion and ductus ligation were performed using computer-aided design. Computational fluid dynamics was utilized to analyze the hemodynamic parameters of varied models based on the patient-specific anatomy and physiological data. The preservation of the PDA competitively reduced the shunt flow but increased total pulmonary perfusion. The shunt flow and ductal flow collided, causing significant and complicated turbulence in the pulmonary artery where low wall shear stress, high oscillatory shear index, and high relative residence time were distributed. The highest energy loss was found when the PDA was preserved. The preservation of PDA is not recommended during MBTS procedures because it negatively influences hemodynamics. This may lead to pulmonary overperfusion, inadequate systemic perfusion, and a heavier cardiac burden, thus increasing the risk of heart failure. Also, it seems to bring no benefit in terms of reducing the risk for thrombosis.

## Introduction

Pulmonary atresia is a complex congenital heart defect characterized by flow interruption between the right ventricle and pulmonary artery. The ductus arteriosus is the fetal blood vessel between the aorta and pulmonary artery, and it can provide pulmonary blood flow postnatally if kept open. Pulmonary perfusion is dependent on the patent ductus arteriosus (PDA) in some patients with pulmonary atresia (Reddy and Hanley, 2012). However, the pulmonary perfusion can still be insufficient due to limited ductal flow. Thus, the modified Blalock–Taussig shunt (MBTS) is required to increase pulmonary blood flow. A Gore-Tex conduit is surgically interposed between the innominate artery (or subclavian artery) and the pulmonary artery to direct blood flow from the systemic circulation to pulmonary circulation (de Leval et al., 1981).

The question of whether to preserve the PDA when performing MBTS procedures in patients with ductal-dependent pulmonary blood flow is still controversial. Surgeons usually make decisions according to experience because there is a lack of studies to ascertain which procedure is better. One reason to preserve the PDA is that it can provide surgeons with additional time to do shunt revision and save lives when the shunt fails due to shunt thrombosis or obstruction. However, the coexisting PDA may be a dangerous flow source for the pulmonary circulation, resulting in pulmonary overperfusion and diastolic runoff of systemic circulation (El-Rassi et al., 2012). These two sources increase the difficulty in regulating the pulmonary blood flow during the perioperative period (Dirks et al., 2013).

Computational fluid dynamics (CFD) and computer-aided design (CAD) enable quantitative investigations of hemodynamic characteristics to find the optimal surgery design (Liu et al., 2014; Celestin et al., 2015; Piskin et al., 2017). Esmaily-Moghadam et al. (2015) utilized the idealized vascular models to explore the hemodynamics of PDA and MBTS and concluded that MBTS with the preservation of PDA did not bring benefits in oxygen delivery but increased the risk of thrombosis. This research is limited in clinical practice due to not considering the patient-specific anatomy. Zhang et al. (2020) also investigated the effects of PDA on MBTS based on the medical images of the patient and the virtually designed PDA. However, the study by Zhang did not give a definite conclusion about the preservation or ligation of PDA but emphasized the importance of considering individual patient conditions in the surgical decision-making process. This study sought to analyze the hemodynamic characteristics of MBTS (with and without the PDA) based on the patient-specific anatomy and physiological conditions. The flow distribution, velocity streamlines, wall shear stress (WSS), oscillatory shear index (OSI), relative residence time (RRT), and energy loss (EL) were analyzed using CFD. This was performed to understand the flow features and to aid in preoperational surgery design and prediction of postoperative prognosis.

## Materials and Methods

### Clinical Data Acquisition

For this study, a 2-month-old female child born with pulmonary atresia and PDA was included. Informed consent was obtained from the guardians of the patient, and the local institutional review board together with the regional research ethics committee of the Shanghai Children's Medical Center (SCMC) affiliated to the Shanghai Jiao Tong University School of Medicine approved all associated studies. Patient-specific medical images were acquired by a 64-slice spiral CT scanner (GE Discovery CT750 HD, USA) before surgery. The slice thickness was 0.625 mm, and each view was of a 512 × 512 pixel field. The echocardiographic data were obtained simultaneously.

### Model Reconstruction and Virtual Surgery

The three-dimensional (3D) vascular model (Model 1) was reconstructed by Materialise® Mimics Innovation Suite 20.0 (Materialise NV, Leuven, Belgium) based on the patient-specific CT images. The PDA has an irregular shape, as its diameter reduces between the end of the aorta and the end of the pulmonary artery. There is an expanded lumen in the confluent site of the left pulmonary artery (LPA) and right pulmonary artery (RPA). CAD was utilized to perform virtual surgeries.

**Figure 1:**
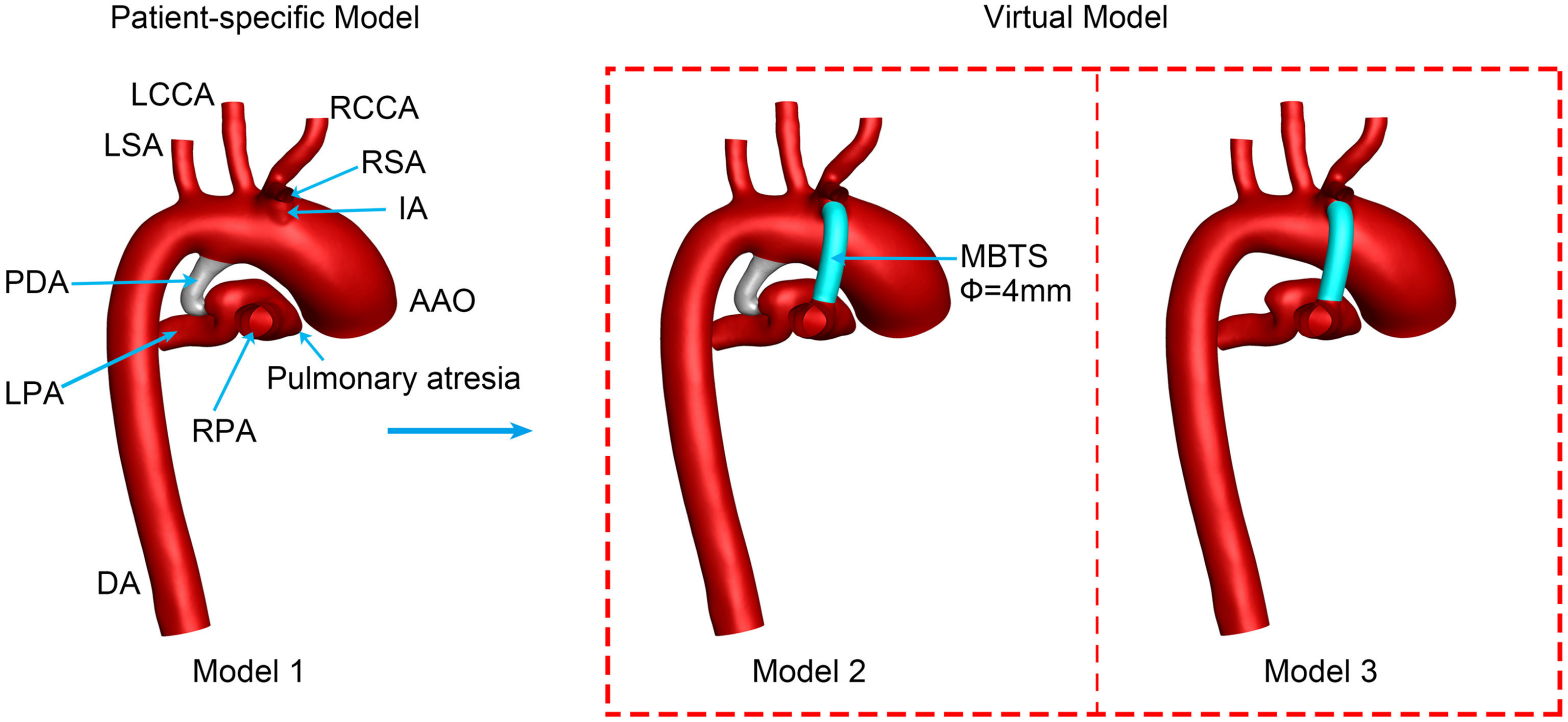
Three-dimensional vascular models. PDA, patent ductus arteriosus; MBTS, modified Blalock-Taussig shunt; AAO, ascending aorta; DA, descending aorta; RSA, right subclavian artery; RCCA, right common carotid artery; RPA, right pulmonary artery; LCCA, left common carotid artery; LSA, left subclavian artery; LPA, left pulmonary artery; Φ, diameter.

A 4-mm conduit was selected to connect the innominate artery (IA) and RPA according to the suggestions of the surgeon. The newly created model was named Model 2, and the PDA of Model 2 was preserved. Model 3 was created based on Model 2, with the PDA being wholly removed to mimic the surgical ligation of PDA. Figure 1 displays the three models in detail.

### Numerical Simulation

#### Governing Equations

The blood was regarded as a Newtonian fluid, and the 3D incompressible Navier–Stokes equation was solved to simulate the blood flow of the shunt domain (Qian et al., 2010), shown as follows:

(1) $$\begin{array}{l} \frac{\partial \left(\rho u_{i}\right)}{\partial t}+\frac{\partial \left(\rho u_{i}u_{j}\right)}{\partial x_{j}}=-\frac{\partial p}{\partial x_{i}}+\frac{\partial }{\partial x_{j}}\left[\mu \left(\frac{\partial u_{i}}{\partial x_{j}}+\frac{\partial u_{j}}{\partial x_{i}}\right)\right]+f_{i} \end{array}$$

(2) $$\begin{array}{l} \frac{\partial \rho }{\partial t}+\frac{\partial \left(\rho u_{j}\right)}{\partial x_{j}}\;=\;0 \end{array}$$

where *i, j* = 1, 2, 3, *x*_1_, *x*_2_, and *x*_3_ represent coordinate axes; *u*_*i*_ and *u*_*j*_ are velocity vectors; *p* is pressure; μ is viscosity; ρ is density; and *t* is time. The term *f*_*i*_ means the action of body forces. The standard *k*–ε turbulence model was applied (Qian et al., 2010).

#### Mesh Generation

Three models were discretized by ANSYS®-ICEM CFD 2020 (ANSYS Inc., USA) for calculation. The tetrahedral mesh was fabricated in the interior, and five layers of prism mesh were generated along the vascular wall in the near-wall region to make calculated parameters more accurate. The inlet was extruded with a length 20 times the inlet diameter to make the inflow fully developed. Each outlet was lengthened 60 times the outlet diameter to simulate the peripheral vasculature for developing antegrade flow. Grid-independent verification was performed using Model 1, and the EL was set as the indicator with the number of grids changing. The results indicated that a grid number of around 1 million would generate the most efficient mesh. Grid numbers of all models (Table 1) were more than 1 million, which were sufficient to produce robust calculations in this study.

**Table 1:** Mesh information.

**Model**	**Total nodes**	**Total elements**
Model 1	542,760	1,336,519
Model 2	554,755	1,370,829
Model 3	513,635	1,256,730

#### Boundary Conditions

**Figure 2:**
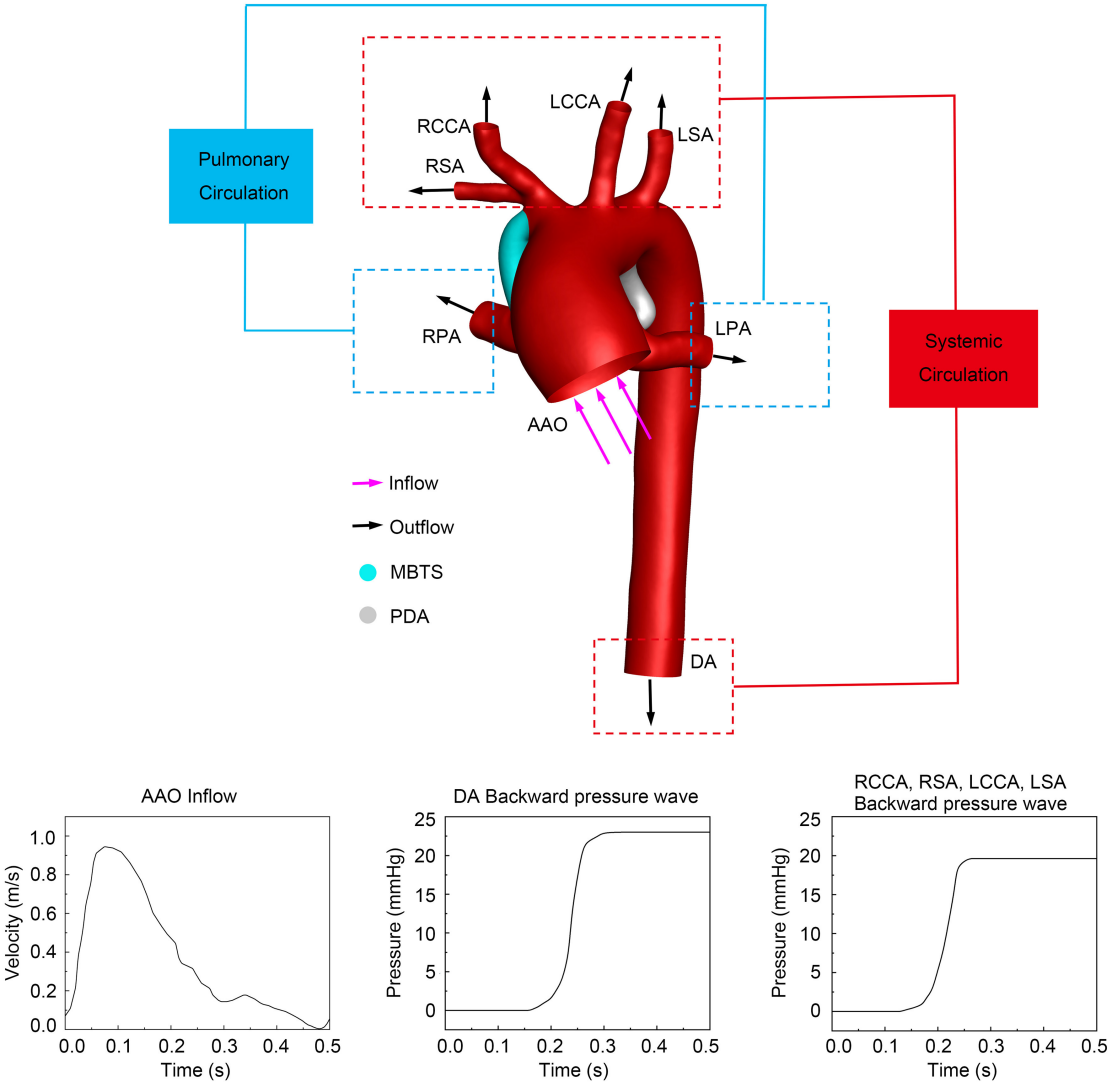
The computational system and boundary conditions. The purple arrows represent the direction of inflow, and the black arrows indicate the direction of outflow. The inlet pulsatile velocity and the backward pressure waves in different outlets are shown at the bottom. PDA, patent ductus arteriosus; MBTS, modified Blalock-Taussig shunt; AAO, ascending aorta; DA, descending aorta; RSA, right subclavian artery; RCCA, right common carotid artery; RPA, right pulmonary artery; LCCA, left common carotid artery; LSA, left subclavian artery; LPA, left pulmonary artery.

The ascending aorta (AAO) was the only inlet, and the inflow was the pulsatile velocity of AAO measured by the real-time echocardiography (Figure 2). A reference pressure of 50 mmHg (arterial blood pressure at end-diastole) was imposed on the aorta domain. The outlets included the descending aorta (DA), right subclavian artery (RSA), right common carotid artery (RCCA), left common carotid artery (LCCA), left subclavian artery (LSA), LPA, and RPA. Previous literature suggested that the measured pressure wave is composed of the forward pressure wave and the backward pressure wave, to explain the pressure wave propagation (Parker and Jones, 1990; Khir et al., 2001; Trachet et al., 2010). The forward pressure wave is derived from the inflow ejected by ventricle contraction. The backward pressure wave is the reflected wave formed by the downstream artery branches. To make the simulation close to the physiology, the backward pressure wave was considered and applied in DA and other aortic branches (Liu et al., 2011, 2015), as is shown in Figure 2. Because the diameters of aortic branched vessels were similar, the backward pressure waves of four outlets (RCCA, RSA, LCCA, and LSA) were assumed the same. A constant pressure of 10 mmHg was simultaneously utilized in the LPA and RPA (Sundareswaran et al., 2006; Sun et al., 2014). Rigid and impermeable vascular walls with no slip were assumed. The same boundary conditions were set in the three models to compare the hemodynamic characteristics of the preservation of PDA or ligation during MBTS procedures.

#### Simulations

ANSYS®-Fluent 2020 (ANSYS Inc., USA) was used to finish the transient simulations of three models. The semi-implicit (SIMPLE) method and the second-order upwind scheme were applied. Because the blood flow in large arteries is pulsatile, high-speed, and high-shear and the diameter of the aorta is much larger than the diameter of a single red blood cell, the blood could be simplified as a Newtonian fluid with a constant density of 1,060 kg·m^−3^ and viscosity of 4.0 × 10^−3^ Pa·s (Liu et al., 2015; Ceballos et al., 2019). The time step was 10^−5^ s, and the convergence criterion was 10^−5^. Three cardiac cycles were calculated, and the results of the third cycle were analyzed. The computational analysis system (currently used) was described in detail and validated in previous studies (Qian et al., 2010; Liu et al., 2011, 2015).

#### Hemodynamic Parameters

The flow rates, pulmonary/systemic ratio (Q_P_/Q_S_), and the LPA/RPA ratio (Q_LPA_/Q_RPA_) were calculated to evaluate the flow interactions and distribution.

The WSS is considered to be related to vascular endothelial injuries and thrombosis (Koskinas et al., 2012; Kumar et al., 2018), calculated according to the equation as follows:

(3) $$\begin{array}{l} \tau_{w}={-\mu \;\frac{\partial u_{x}}{\partial y}|}_{y\;=\;0} \end{array}$$

where *u*_*x*_ is the velocity of fluid near the vessel wall and *y* is the distance to the vessel wall.

The time-averaged WSS (TAWSS) is defined as follows:

(4) $$\begin{array}{l} TAWSS\;=\;\frac{1}{T}\int_{0}^{T}|\tau_{w}|dt \end{array}$$

where *T* is the time of a cardiac cycle, equal to 0.5 s.

The OSI is a non-dimensional metric to describe the WSS vector deflection from the predominant direction of blood flow over a cardiac cycle (Ku et al., 1985; He and Ku, 1996) and is defined as follows:

(5) $$\begin{array}{l} OSI\;=\;\frac{1}{2}\left[1-\frac{\left|\int_{0}^{T}\tau_{w}dt\right|}{\int_{0}^{T}|\tau_{w}|dt}\right] \end{array}$$

The RRT is the residence time that the particles spent around the endothelium, introduced as inversely proportional to OSI and TAWSS (Himburg et al., 2004),

(6) $$\begin{array}{l} RRT\sim \frac{1}{\left(1-2\times OSI\right)\times TAWSS} \end{array}$$

The EL is a quantitative indicator to evaluate hemodynamic efficiency and cardiac workload, given by the equation as follows:

(7) $$\begin{array}{l} \;EL\;=\;E_{inlet}\;-\;E_{outlet}\\ =\underset{inlet}{\sum }\left(p_{in}+\frac{1}{2}\rho {V_{in}}^{2}\right)Q_{in}\;-\underset{outlet}{\sum }\left(p_{out}+\frac{1}{2}\rho {V_{out}}^{2}\right)Q_{out} \end{array}$$

where *V* and *Q* are velocity and flow rate, respectively, *in* indicates the inlet, and *out* indicates the outlet.

## Results

### Flow Distribution

Blood flow from the right ventricle to the pulmonary artery was completely obstructed in the subject of the study. Therefore, pulmonary perfusion was provided by the flow through the MBTS and/or the PDA. Model 2 with coexisting MBTS and PDA was associated with the highest pulmonary perfusion and Q_P_/Q_S_, as listed in Table 2. The pulmonary perfusion of Model 1 was the lowest. The shunt flow of Model 2 was lower than that of Model 3, and the ductal flow of Model 2 was lower than that of Model 1. It turns out that competitive flow was present when the PDA was preserved. The Q_LPA_/Q_RPA_ of Model 2 was located between Model 1 and Model 3, and the difference of Q_LPA_/Q_RPA_ was insignificant among the three models. The flow distribution between the LPA and RPA was asymmetric and tended to RPA in all models.

**Table 2:** Flow distribution.

**Model**	**Q_**MBTS**_ (L/min)**	**Q_**PDA**_ (L/min)**	**Q_**P**_ (L/min)**	**Q_**S**_ (L/min)**	**Q_**P**_/Q_**S**_**	**Q_**LPA**_ (L/min)**	**Q_**RPA**_ (L/min)**	**Q_**LPA**_/Q_**RPA**_**
Model 1	/	0.91	0.91	3.43	0.27	0.35	0.56	0.63
Model 2	1.83	0.73	2.56	1.78	1.44	0.97	1.59	0.61
Model 3	1.94	/	1.94	2.40	0.81	0.71	1.23	0.58

*Q_MBTS_ and Q_PDA_ are flow rates through the modified Blalock–Taussig shunt and the patent ductus arteriosus. Q_P_, Q_S_, Q_LPA_, and Q_RPA_ are flow rates to the pulmonary circulation, systemic circulation, left pulmonary artery, and right pulmonary artery, respectively*.

### Velocity Streamlines

**Figure 3:**
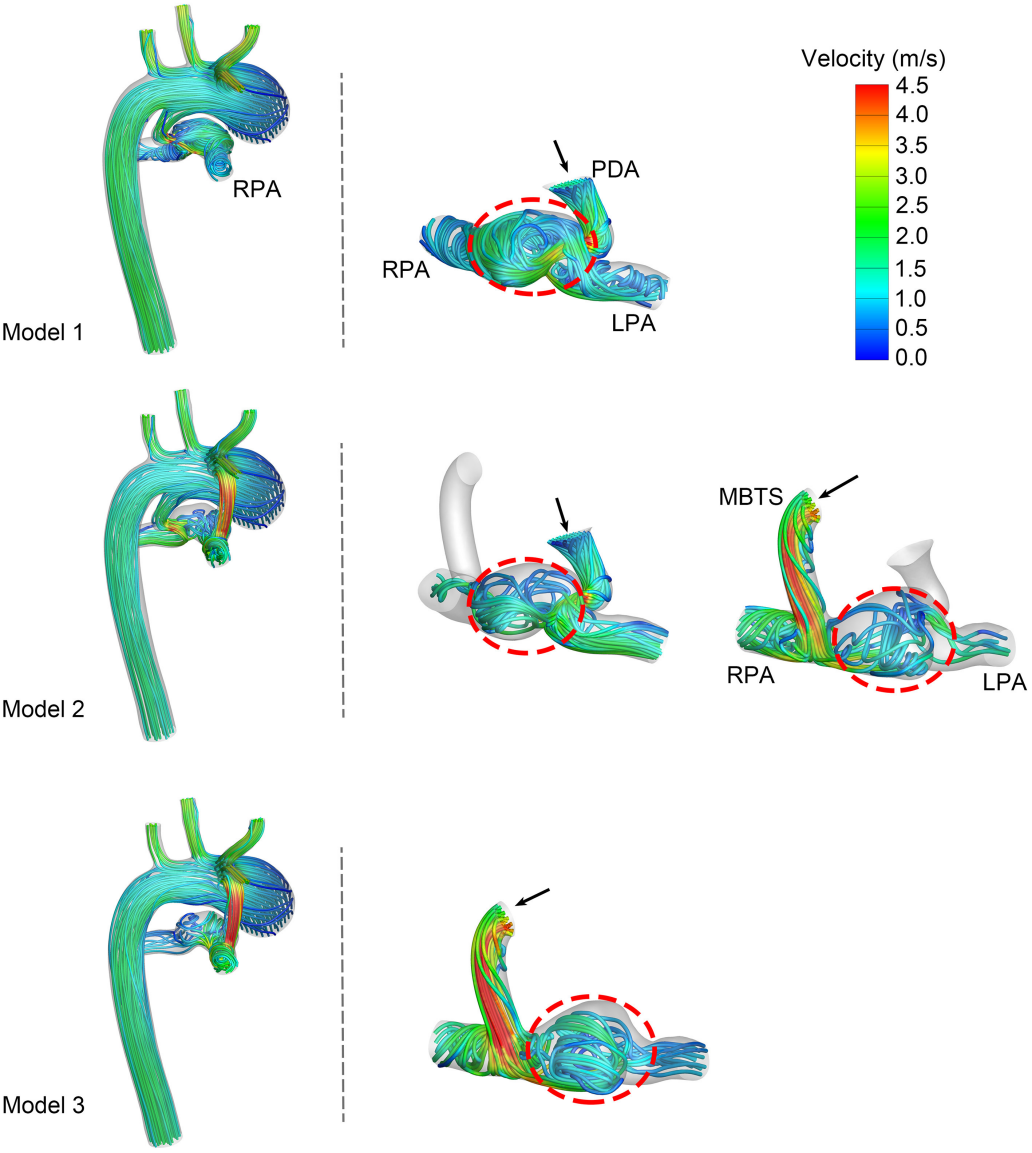
Velocity streamlines at the systolic peak. The left column exhibits the velocity streamlines of the whole model. Another two columns present the streamlines through the patent ductus arteriosus (PDA) and modified Blalock-Taussig shunt (MBTS). The black arrows express the direction of flow, and turbulence is circled in a red dashed line. LPA, left pulmonary artery; RPA, right pulmonary artery.

The velocity streamlines at the systolic peak are shown in Figure 3. Shunt flow was associated with high velocity no matter whether the PDA was ligated or not. This shunt flow ran through the vertical conduit and impacted the pulmonary artery wall, causing turbulence in Models 2 and 3. More turbulence was formed when the shunt flow and ductal flow rushed against each other in the pulmonary artery between the MBTS and PDA. Fewer streamlines through the PDA went into the RPA in Model 2 compared with Model 1. Similarly, fewer streamlines through the MBTS went into the LPA in Model 2 compared with Model 3. These results suggested that the flow split between the LPA and RPA was affected by flow interactions between the shunt and ductus.

### Wall Shear Stress

**Figure 4:**
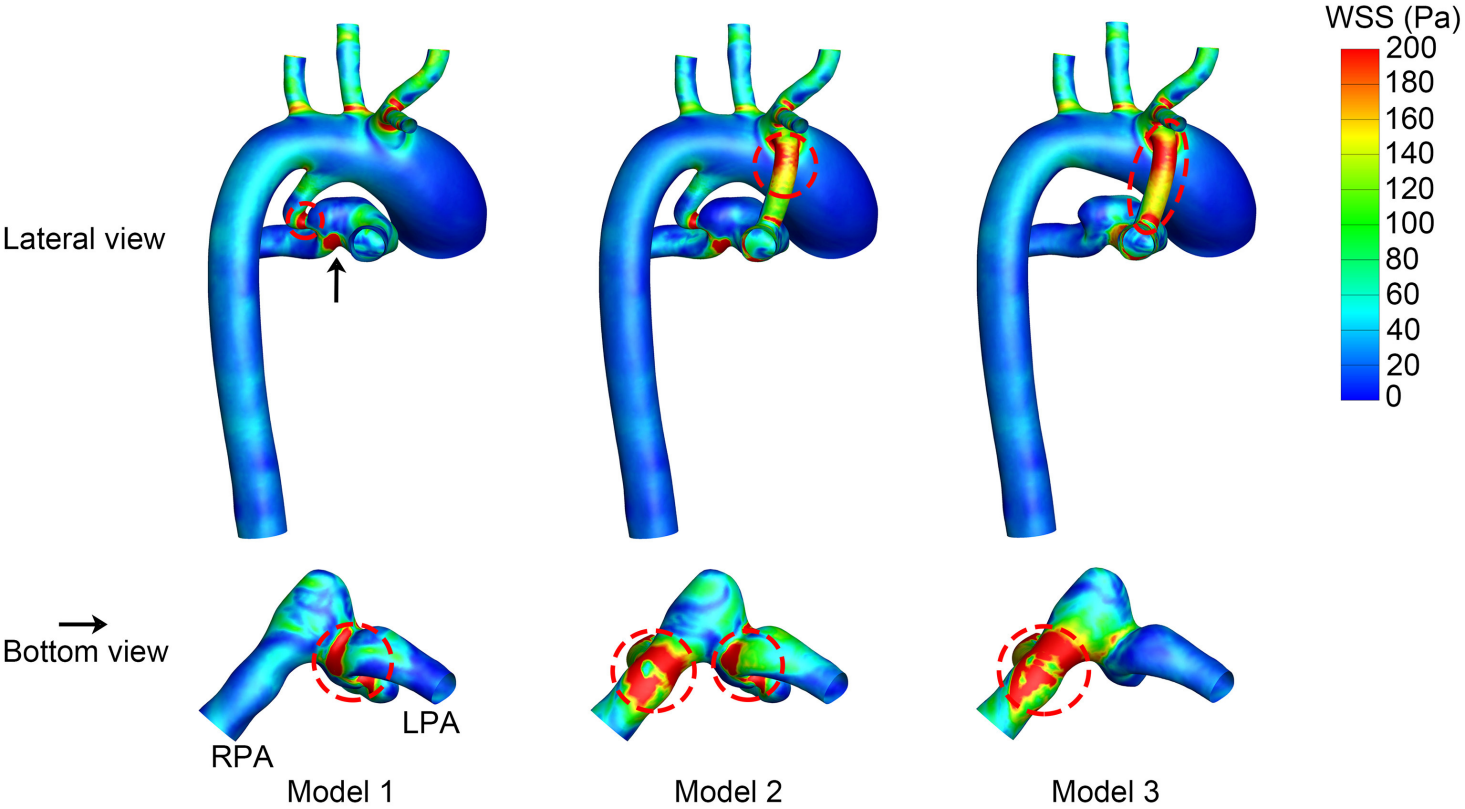
Wall shear stress (WSS) at the systolic peak. The upper row is the lateral view of the whole model, and the lower row is the bottom view of the model except for the aorta. The black arrow indicates the perspective of the bottom view. Areas with high WSS are marked in a red dashed circle. LPA, left pulmonary artery; RPA, right pulmonary artery.

**Figure 5:**
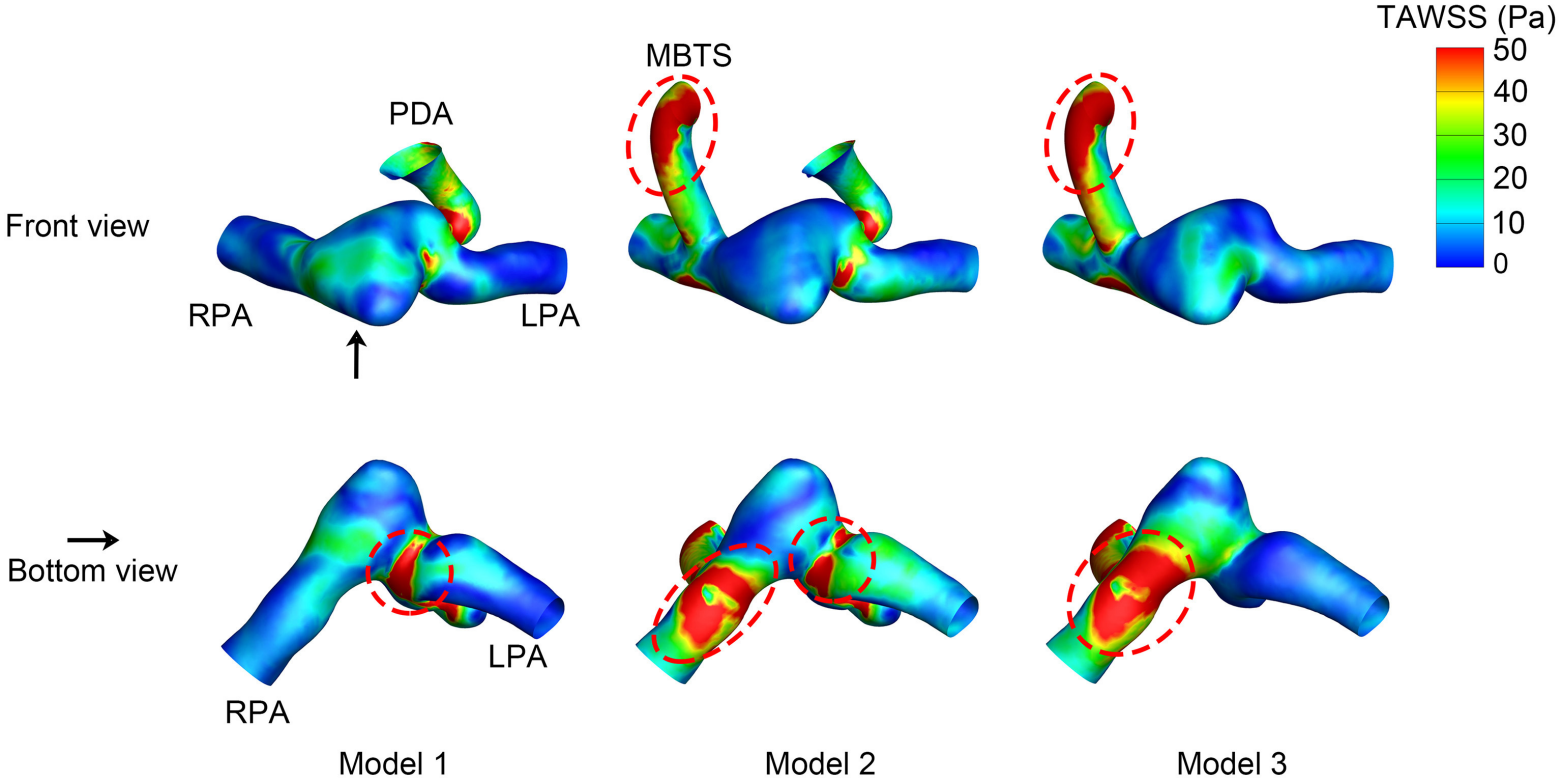
Time-averaged wall shear stress (TAWSS). The upper row is the front view, and the lower row is the bottom view of each model except for the aorta. The black arrow indicates the perspective of the bottom view. PDA, patent ductus arteriosus; MBTS, modified Blalock-Taussig shunt; LPA, left pulmonary artery; RPA, right pulmonary artery.

The WSS distribution at the systolic peak is shown in Figure 4. The shunts of Model 2 and Model 3 were associated with high WSS. The pulmonary anastomotic site of the shunt was found with sharp WSS distribution compared with other sites of the pulmonary artery. High WSS was also found in the confluent site of the PDA and pulmonary artery, especially evident in Model 1. The TAWSS was also higher in the shunt and the anastomosed site of the pulmonary artery (Figure 5). In the pulmonary artery between the MBTS and PDA, the TAWSS was notably lower in Model 2.

### Oscillatory Shear Index

**Figure 6:**
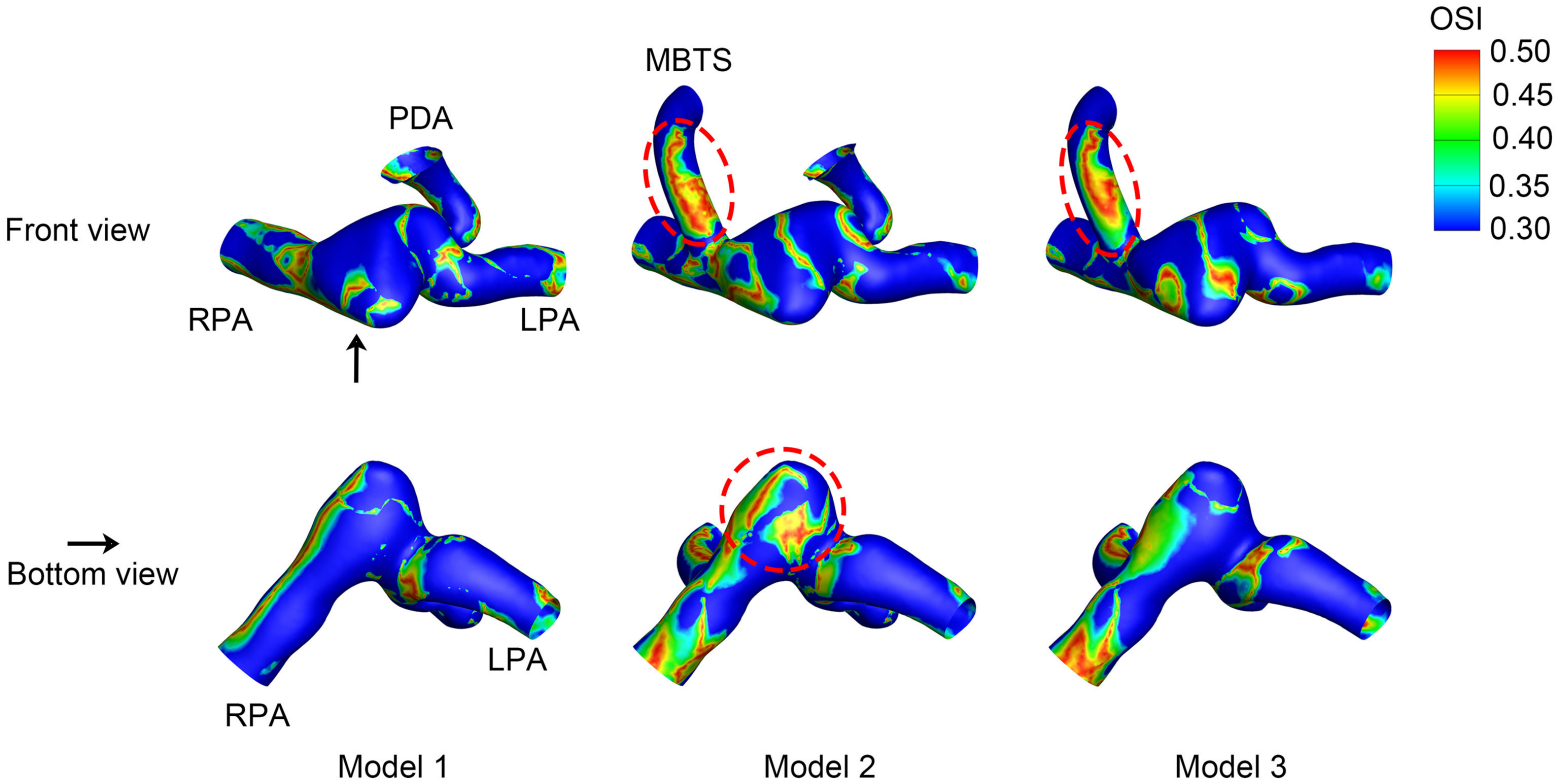
Oscillatory shear index (OSI). The upper row is the front view, and the lower row is the bottom view of each model except for the aorta. The black arrow indicates the perspective of the bottom view. PDA, patent ductus arteriosus; MBTS, modified Blalock-Taussig shunt; LPA, left pulmonary artery; RPA, right pulmonary artery.

The OSI value ranged from 0 to 0.5, i.e., 0 indicated non-cyclic variation and 0.5 indicated 180.0° deflection from the WSS direction. A high OSI was found in the shunt of Models 2 and 3 (Figure 6). The area of high OSI distributed in the shunt of Model 2 was larger. The pulmonary artery between the MBTS and PDA was also associated with higher OSI. The maximum OSI reached 0.49, which was more profound in Model 2.

### Relative Residence Time

**Figure 7:**
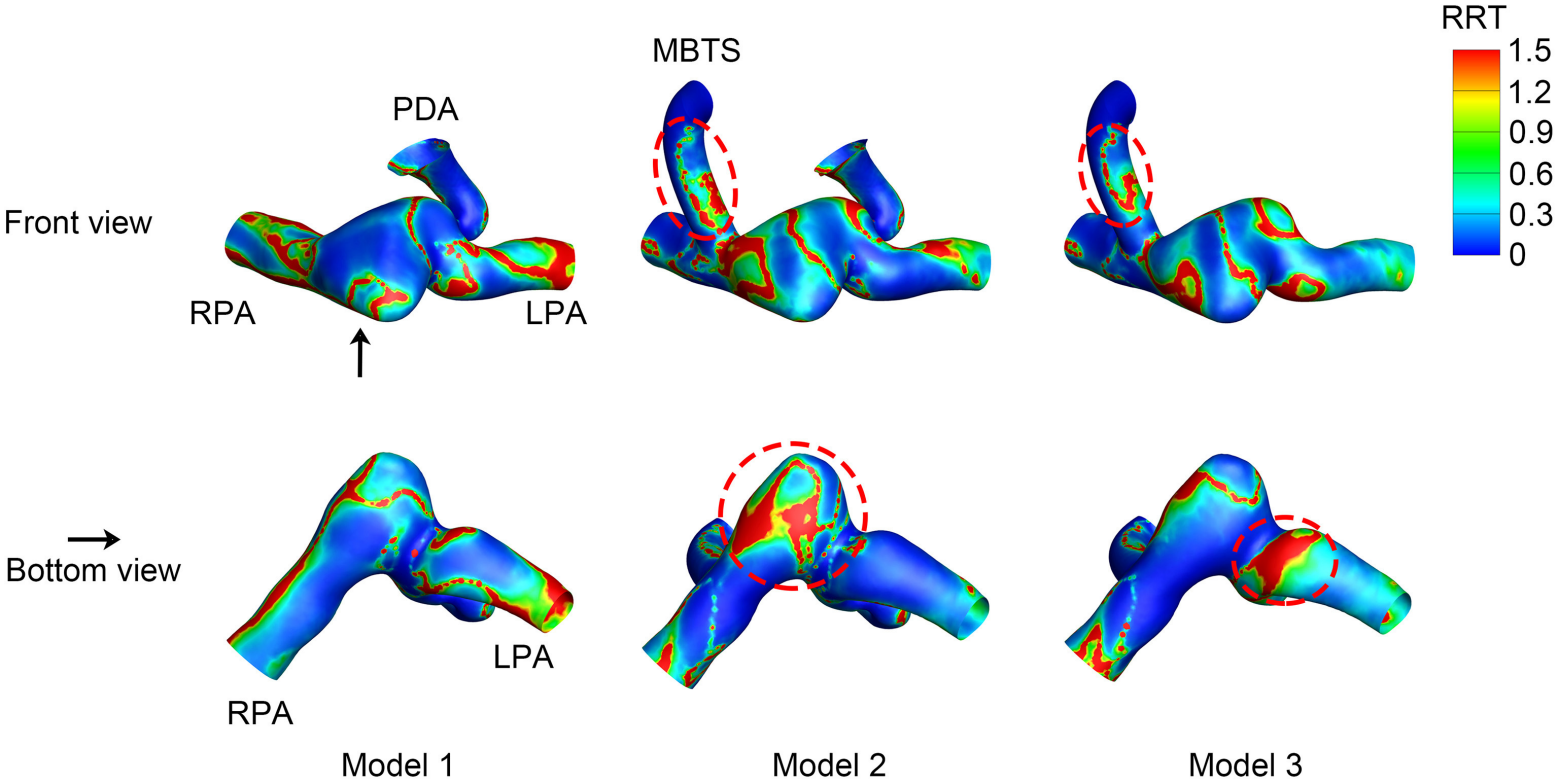
Relative residence time (RRT). The upper row is the front view, and the lower row is the bottom view of each model except for the aorta. The black arrow indicates the perspective of the bottom view. PDA, patent ductus arteriosus; MBTS, modified Blalock-Taussig shunt; LPA, left pulmonary artery; RPA, right pulmonary artery.

The RRT distribution varied among the three models. The inner side of the shunt was associated with high RRT (Figure 7). High RRT mainly appeared downstream of the pulmonary artery in Model 1. As for Model 2, the pulmonary artery between the MBTS and PDA was associated with high RRT. The area of high RRT distribution was smaller in Model 3 compared with Model 2.

### Energy Loss

**Figure 8:**
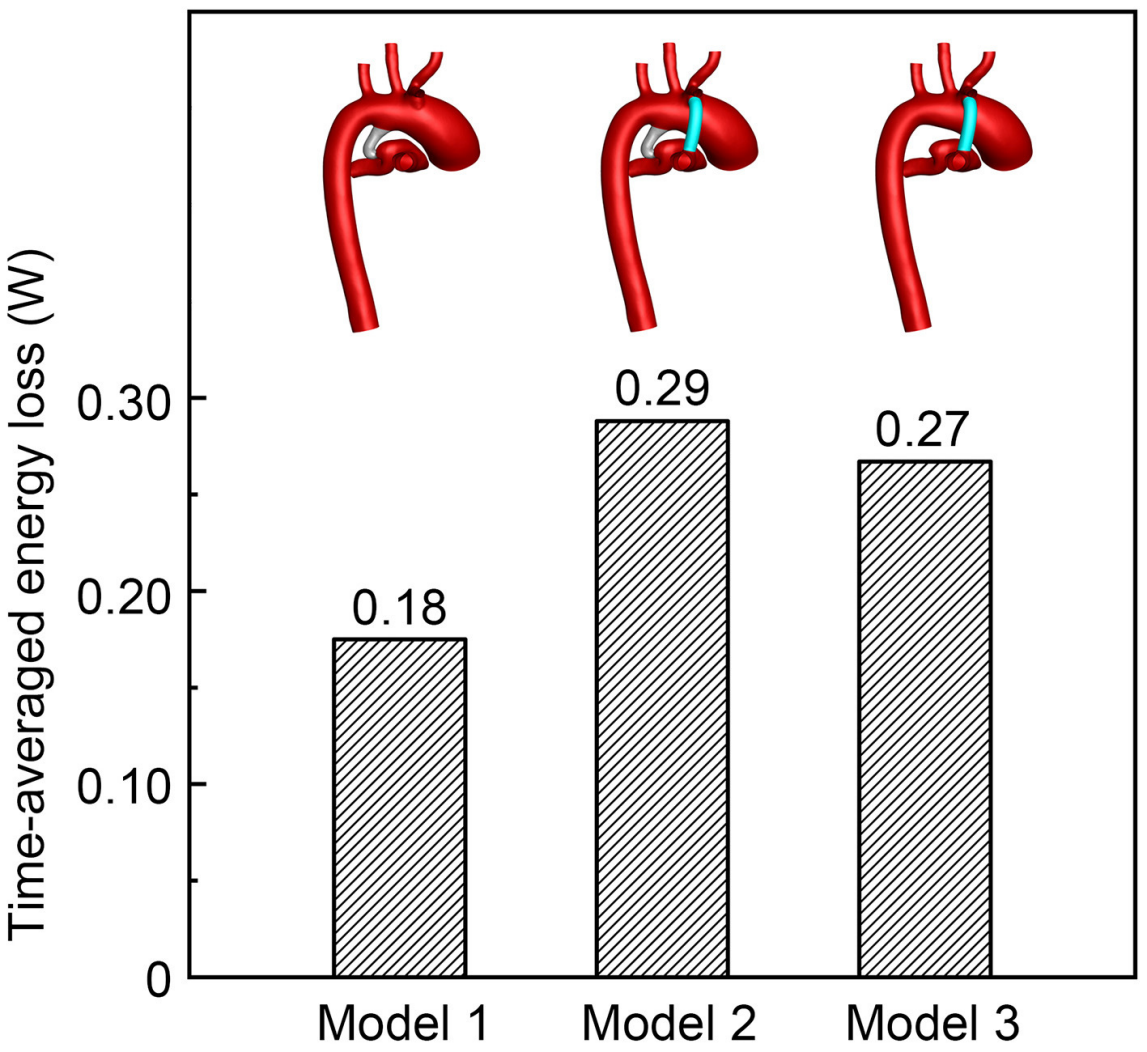
Time-averaged energy loss of a cardiac cycle.

The EL is used to evaluate energy efficiency and ventricular workload quantitatively. Figure 8 shows the time-averaged EL through a cardiac cycle. The EL of Model 2 was the highest, and the preservation of PDA increased energy dissipation of flow and cardiac workload.

## Discussion

In the patient with pulmonary atresia, the pulmonary blood flow comes from the systemic circulation through the PDA and/or surgically created MBTS. Based on the patient-specific anatomy and physiological data, the simulation results demonstrated the existence of flow competition between the MBTS and PDA and the influences of flow interactions. This provided quantitative and qualitative hemodynamic parameters to help with surgery design and postoperative prediction.

Balanced flow distribution between the pulmonary circulation and systemic circulation is intimately related to the prognosis of MBTS, which can be quantitatively evaluated by Q_P_/Q_S_. Excessive pulmonary blood flow may lead to pulmonary overperfusion and diastolic runoff of the systemic circulation. Insufficient pulmonary perfusion would cause inadequate oxygen delivery, thrombosis, shunt occlusion, and an underdeveloped pulmonary artery (Fenton et al., 2003; Dirks et al., 2013). The ideal value of Q_P_/Q_S_ is slightly <1, which helps provide adequate pulmonary blood flow and oxygen delivery without reducing systemic perfusion and myocardial blood flow (Barnea et al., 1994, 1998). However, if the value of Q_P_/Q_S_ is <0.7, a further decrease of Q_P_/Q_S_ reduces the oxygen delivery and negatively affects hemodynamics (Riordan et al., 1996). Since the same boundary conditions were applied in simulations, the values of Q_P_/Q_S_ were mainly affected by the anatomy. The Q_P_/Q_S_ of Model 1 was <0.7, suggesting that the PDA alone cannot provide enough pulmonary perfusion, and this was the reason for cyanosis and corresponded with the diagnosis of the patient. The Q_P_/Q_S_ of Model 3 was between 0.7 and 1, providing sufficient pulmonary perfusion without resulting in insufficient systemic blood flow. The Q_P_/Q_S_ of Model 2 was more than 1, increasing the risk of pulmonary overperfusion and congestive heart failure.

Balanced flow distribution between the LPA and RPA benefits symmetric pulmonary artery growth in patients with bilateral hypoplastic pulmonary arteries. Coexisting MBTS and PDA produced a value of Q_LPA_/Q_RPA_ located between Model 1 and Model 3. The Q_LPA_/Q_RPA_ differences were insignificant among the three models, and the pulmonary flow favored RPA in all models. The unimportant differences could be explained by the patient-specific morphology of the pulmonary artery because the boundary conditions of LPA and RPA were assumed to be similar in this study. The RPA is associated with a larger diameter, and the confluent site of LPA and RPA has an expanded lumen. The swirling flow was developed in the confluent pulmonary artery despite the PDA ligation or MBTS insertion, which may be the reason for the decreased left pulmonary perfusion. These results are not in line with the study by Zhang using the ideal vascular model (Zhang et al., 2020). This study reflected the concerning influence of the individualized pulmonary vascular geometry on the flow field and shed light on the importance of numerical simulation based on the patient-specific anatomy.

The hemodynamic EL is an effective indicator caused by blood movement, flow collision, and turbulence to evaluate energy efficiency and ventricular workload quantitatively. The MBTS with PDA was associated with the largest EL, which could be explained by augmented ventricular volume load, flow interactions, and significant turbulence. As time accumulates, coexisting MBTS and PDA will gradually increase cardiac workload. The EL of Model 3 was higher than Model 1, suggesting that the MBTS increased the cardiac burden. Adequate coronary perfusion is critical for heart pump with an augmented cardiac burden. Given that coronary perfusion decreases as pulmonary perfusion increases, the preservation of PDA may augment the risk of heart failure in neonates with hypoplastic myocardium.

Shunt-related complications such as thrombosis and occlusion remain unresolved. Very low or very high WSS is likely to cause endothelial injury and platelet activation, increasing the risk of thrombosis and occlusion (Holme et al., 1997; Kumar et al., 2018). Low WSS and high OSI are associated with endothelial injury and atherosclerosis, and OSI > 0.2 is a risk factor for atherosclerosis (Frydrychowicz et al., 2009). RRT is used to assess the time that the particles spend in the endothelium, and high RRT indicates a higher possibility of particle deposition. The outer sides of the shunt and anastomosed sites in the pulmonary artery were associated with higher WSS. Low WSS and high OSI were developed in the inner side of the shunt. Competitive flow also yielded a region with lower WSS, higher OSI, and RRT inside the pulmonary artery. These results suggest that the preservation of PDA brings no benefits in potential risks of shunt thrombosis and occlusion, which still need to be carefully monitored during the perioperative period and the long-term follow-up.

There were several limitations in this study. First, the assumption of a rigid wall without considering the elasticity and compliance of the great vessels. Although fluid–structure interaction can be applied to consider the elasticity, the rigid wall can achieve comparative results with minor errors (Esmaily-Moghadam et al., 2015). The pressure wave reflection was set in all outlets to reduce the influence of the rigid wall assumption, which was validated in previous studies (Liu et al., 2011, 2015). Second, only one type of shunt design was discussed in this study. Shunt diameter, shunt position, anastomosis angle, and shunt shape will affect the flow features (Piskin et al., 2017). Nevertheless, the shunt design in this study was appropriate to be applied. According to the advice of the surgeon, a shunt size of 4 mm was selected, and the MBTS was placed in the contralateral side of the PDA. The anastomotic sites were decided based on the spatial position and geometry of the aorta and pulmonary artery branches. Third, the position and size of PDA affect the flow features of MBTS. This study preliminarily investigated the effects of the existence of PDA on the shunt flow. A further study focusing on the effects of the position and morphology of PDA on hemodynamics will be added in the future.

## Conclusion

The MBTS with PDA is accompanied by more pulmonary perfusion, flow competition, large-scale and complicated turbulence, and higher EL. The ligation of PDA is recommended during the MBTS procedure since the preservation of PDA is riskier, and it may lead to pulmonary overperfusion, insufficient systemic blood flow, and a heavier cardiac burden. Low WSS, high OSI, and high RRT are found in the pulmonary artery between the MBTS and PDA when the PDA is preserved, which does not reduce the risk of thrombosis. The risks of shunt thrombosis and occlusion are still worth noting in the perioperative period and the long-term follow-up. Vascular morphology plays a vital role in hemodynamics, which should be considered in surgery design.

## Data Availability Statement

The original contributions presented in the study are included in the article, further inquiries can be directed to the corresponding authors.

## Ethics Statement

Written informed consent was obtained from the guardians of the patient. All associated studies were approved by the local institutional review board and regional research ethics committee of Shanghai Children's Medical Center (SCMC) affiliated to Shanghai Jiao Tong University School of Medicine.

## Author Contributions

JX was responsible for numerical simulation, data analysis, and manuscript preparation. QS was responsible for the clinical data acquirement and virtual surgery. YQ developed the idea of the study and assisted in the manuscript review. LH assisted in acquiring clinical images. ZT assisted in the reconstruction of the 3D vascular model. JinfL was responsible for data analysis and manuscript review. JinlL conceived the study and helped with manuscript revision. All authors contributed to the manuscript and approved the submitted version.

## Conflict of Interest

The authors declare that the research was conducted in the absence of any commercial or financial relationships that could be construed as a potential conflict of interest.

## Publisher's Note

All claims expressed in this article are solely those of the authors and do not necessarily represent those of their affiliated organizations, or those of the publisher, the editors and the reviewers. Any product that may be evaluated in this article, or claim that may be made by its manufacturer, is not guaranteed or endorsed by the publisher.

## References

[B1] Barnea O. Austin E. H. Richman B. Santamore W. P. (1994). Balancing the circulation: theoretic optimization of pulmonary/systemic flow ratio in hypoplastic left heart syndrome. J. Am. Coll. Cardiol. 24, 1376–1381. 10.1016/0735-1097(94)90123-6 7523473

[B2] Barnea O. Santamore W. P. Rossi A. Salloum E. Chien S. Austin E. H. (1998). Estimation of oxygen delivery in newborns with a univentricular circulation. Circulation 98, 1407–1413. 10.1161/01.CIR.98.14.1407 9760295

[B3] Ceballos A. Prather R. Divo E. Kassab A. J. DeCampli W. M. (2019). Patient-specific multi-scale model analysis of hemodynamics following the hybrid Norwood procedure for hypoplastic left heart syndrome: effects of reverse Blalock-Taussig shunt diameter. Cardiovasc. Eng. Technol. 10, 136–154. 10.1007/s13239-018-00396-w 30515683

[B4] Celestin C. Guillot M. Ross-Ascuitto N. Ascuitto R. (2015). Computational fluid dynamics characterization of blood flow in central aorta to pulmonary artery connections: importance of shunt angulation as a determinant of shear stress-induced thrombosis. Pediatr. Cardiol. 36, 600–615. 10.1007/s00246-014-1055-7 25404555

[B5] de Leval M. R. McKay R. Jones M. Stark J. Macartney F. J. (1981). Modified Blalock-Taussig shunt. Use of subclavian artery orifice as flow regulator in prosthetic systemic-pulmonary artery shunts. J. Thorac. Cardiovasc. Surg. 81, 112–119. 10.1016/S0022-5223(19)37668-8 6450303

[B6] Dirks V. Pretre R. Knirsch W. Valsangiacomo Buechel E. R. Seifert B. Schweiger M.. (2013). Modified Blalock Taussig shunt: a not-so-simple palliative procedure. Eur. J. Cardiothorac. Surg. 44, 1096–1102. 10.1093/ejcts/ezt172 23539419

[B7] El-Rassi I. Soueide A. Chabb B. (2012). Blalock-Taussig shunts with and without closure of the ductus arteriosus. Ann. Thorac. Surg. 93, 1399–1400. 10.1016/j.athoracsur.2011.10.052 22450101

[B8] Esmaily-Moghadam M. Murtuza B. Hsia T.-Y. Marsden A. (2015). Simulations reveal adverse hemodynamics in patients with multiple systemic to pulmonary shunts. J. Biomech. Eng. 137, 0310011–03100112. 10.1115/1.4029429 25531794 PMC4321115

[B9] Fenton K. N. Siewers R. D. Rebovich B. Pigula F. A. (2003). Interim mortality in infants with systemic-to-pulmonary artery shunts. Ann. Thorac. Surg. 76, 152–156. 10.1016/S0003-4975(03)00168-1 12842531

[B10] Frydrychowicz A. Stalder A. F. Russe M. F. Bock J. Bauer S. Harloff A.. (2009). Three-dimensional analysis of segmental wall shear stress in the aorta by flow-sensitive four-dimensional-MRI. J. Magn. Reson. Imaging 30, 77–84. 10.1002/jmri.21790 19557849

[B11] He X. Ku D. N. (1996). Pulsatile flow in the human left coronary artery bifurcation: average conditions. J. Biomech. Eng. 118, 74–82. 10.1115/1.2795948 8833077

[B12] Himburg H. A. Grzybowski D. M. Hazel A. L. LaMack J. A. Li X.-M. Friedman M. H. (2004). Spatial comparison between wall shear stress measures and porcine arterial endothelial permeability. Am. J. Physiol. Heart Circ. Physiol. 286, H1916–H1922. 10.1152/ajpheart.00897.2003 14715506

[B13] Holme P. A. Orvim U. Hamers M. J. Solum N. O. Brosstad F. R. Barstad R. M.. (1997). Shear-induced platelet activation and platelet microparticle formation at blood flow conditions as in arteries with a severe stenosis. Arterioscler. Thromb. Vasc. Biol. 17, 646–653. 10.1161/01.ATV.17.4.646 9108776

[B14] Khir A. W. O'Brien A. Gibbs J. S. R. Parker K. H. (2001). Determination of wave speed and wave separation in the arteries. J. Biomech. 34, 1145–1155. 10.1016/S0021-9290(01)00076-8 11506785

[B15] Koskinas K. C. Chatzizisis Y. S. Antoniadis A. P. Giannoglou G. D. (2012). Role of endothelial shear stress in stent restenosis and thrombosis: pathophysiologic mechanisms and implications for clinical translation. J. Am. Coll. Cardiol. 59, 1337–1349. 10.1016/j.jacc.2011.10.903 22480478

[B16] Ku D. N. Giddens D. P. Zarins C. K. Glagov S. (1985). Pulsatile flow and atherosclerosis in the human carotid bifurcation. Positive correlation between plaque location and low oscillating shear stress. Arteriosclerosis 5, 293–302. 10.1161/01.ATV.5.3.293 3994585

[B17] Kumar A. Hung O. Y. Piccinelli M. Eshtehardi P. Corban M. T. Sternheim D.. (2018). Low coronary wall shear stress is associated with severe endothelial dysfunction in patients with nonobstructive coronary artery disease. JACC Cardiovasc. Interv. 11, 2072–2080. 10.1016/j.jcin.2018.07.004 30268874 PMC6217963

[B18] Liu J. Sun Q. Hong H. Sun Y. Liu J. Qian Y.. (2015). Medical image-based hemodynamic analysis for modified Blalock–Taussig shunt. J. Mech. Med. Biol. 15:1550035. 10.1142/S0219519415500359

[B19] Liu J. Sun Q. Umezu M. Qian Y. Hong H. Du Z.. (2014). Influence of conduit angles on hemodynamics of modified Blalock-Taussig shunt: computational analysis of patient-specific virtual procedures, in Life System Modeling and Simulation, eds Ma S. Jia L. Li X. Wang L. Zhou H. Sun X. (Berlin, Heidelberg: Springer Berlin Heidelberg), 62–71.

[B20] Liu J. L. Qian Y. Itatani K. Miyakoshi T. Murakami A. Ono M.. (2011). An approach of computational hemodynamics for cardiovascular flow simulation, in ASME-JSME-KSME 2011 Joint Fluids Engineering Conference: Volume 1, Symposia – Parts A, B, C, and D, 1449–1456.

[B21] Parker K. H. Jones C. J. H. (1990). Forward and backward running wave in the arteries: analysis using the method of characteristics. J. Biomech. Eng. 112, 322–326. 10.1115/1.2891191 2214715

[B22] Piskin S. Altin H. F. Yildiz O. Bakir I. Pekkan K. (2017). Hemodynamics of patient-specific aorta-pulmonary shunt configurations. J. Biomech. 50, 166–171. 10.1016/j.jbiomech.2016.11.014 27866675

[B23] Qian Y. Liu J. L. Itatani K. Miyaji K. Umezu M. (2010). Computational hemodynamic analysis in congenital heart disease: simulation of the Norwood procedure. Ann. Biomed. Eng. 38, 2302–2313. 10.1007/s10439-010-9978-5 20195758

[B24] Reddy V. M. Hanley F. L. (2012). Surgical treatment of pulmonary atresia with ventricular septal defect, in Pediatric Cardiac Surgery, eds Mavroudis C. Backer C. Idriss R. F. (UK: Blackwell Publishing Ltd.), 428–442.

[B25] Riordan C. J. Randsbeck F. Storey J. H. Montgomery W. D. Santamore W. P. Austin E. H. 3rd (1996). Effects of oxygen, positive end-expiratory pressure, and carbon dioxide on oxygen delivery in an animal model of the univentricular heart. J. Thorac. Cardiovasc. Surg. 112, 644–654. 10.1016/S0022-5223(96)70047-8 8800151

[B26] Sun Q. Liu J. Qian Y. Zhang H. Wang Q. Sun Y.. (2014). Computational haemodynamic analysis of patient-specific virtual operations for total cavopulmonary connection with dual superior venae cavae. Eur. J. Cardiothorac. Surg. 45, 564–569. 10.1093/ejcts/ezt394 23904133

[B27] Sundareswaran K. S. Kanter K. R. Kitajima H. D. Krishnankutty R. Sabatier J. F. Parks W. J.. (2006). Impaired power output and cardiac index with hypoplastic left heart syndrome: a magnetic resonance imaging study. Ann. Thorac. Surg. 82, 1267–1277. 10.1016/j.athoracsur.2006.05.020 16996919

[B28] Trachet B. Reymond P. Kips J. Swillens A. De Buyzere M. Suys B.. (2010). Numerical validation of a new method to assess aortic pulse wave velocity from a single recording of a brachial artery waveform with an occluding cuff. Ann. Biomed. Eng. 38, 876–888. 10.1007/s10439-010-9945-1 20127171

[B29] Zhang N. Yuan H. Chen X. Liu J. Zhou C. Huang M.. (2020). Hemodynamic of the patent ductus arteriosus in neonates with modified Blalock-Taussig shunts. Comput. Methods Programs Biomed. 186:105223. 10.1016/j.cmpb.2019.105223 31760306

